# Myocardial Viability: Evolving Insights and Challenges in Revascularization and Functional Recovery

**DOI:** 10.3390/jcdd12030106

**Published:** 2025-03-20

**Authors:** Kristoffer Ken Ralota, Jamie Layland, Kyi Thar Han Win, Nay M. Htun

**Affiliations:** 1 Department of Cardiology, Peninsula Health, Frankston, VIC 3199, Australia; ken.ralota@gmail.com (K.K.R.); jlayland@phcn.vic.gov.au (J.L.); k.win@alfred.org.au (K.T.H.W.); 2 Peninsula Clinical School, Monash University, Melbourne, VIC 3800, Australia; 3 Alfred Health, Melbourne, VIC 3004, Australia

**Keywords:** myocardial viability, ischemic cardiomyopathy, heart failure, left ventricular dysfunction, hibernating myocardium, myocardial stunning, cardiac imaging

## Abstract

The prevalence of heart failure, driven significantly by ischemic heart disease, continues to rise globally. Myocardial viability—the potential ability of dysfunctional myocardium to recover contractile function after revascularization—remains an ongoing key area of research in managing ischemic cardiomyopathy. Advances in imaging modalities, including PET/SPECT, cardiac MRI, and dobutamine stress echocardiography, have enabled identification of viable myocardium that can potentially predict their functional recovery following revascularization. Despite these advances, recent evidence from major trials questions the routine reliance on viability testing for revascularization guidance. These studies found a limited correlation between myocardial viability and improved outcomes in key metrics including mortality. Furthermore, they highlighted the effectiveness of guideline-directed medical therapy in improving left ventricular function independent of revascularization. This narrative review explores the concept of myocardial viability, its assessment through contemporary imaging techniques, its clinical utility in decision making for revascularization, and future directions.

## 1. Introduction

Heart failure prevalence among adults in industrialized nations is estimated at 1–3% and is projected to increase [1]. Ischemic heart disease is a leading cause of heart failure contributing to more than half of patients with heart failure and is a significant precipitant of heart failure-related hospitalizations with increased post-discharge mortality [1].

The concept of prolonged myocardial dysfunction in the setting of transient or repetitive ischemia has been known in the literature since the 1980s when Braunwald and Kloner introduced the “hit, run, and stun” concept in their paper [2].

Identifying viable but hypocontractile tissue in patients with ischemic left ventricular (LV) dysfunction is a major clinical interest due to its potential prognostic value and its potential to guide patient selection for revascularization.

Several trials have since investigated whether revascularization improves outcomes in the presence of myocardial viability. The primary objective of this narrative review is to examine evidence from major clinical trials that question the routine reliance on viability testing for guiding revascularization decisions. Additionally, this review explores the concept of myocardial viability, its assessment through contemporary imaging techniques, and potential future directions.

### 1.1. What Is Myocardial Viability?

The ischemic cascade following acute coronary occlusion begins with reversible myocardial contractile dysfunction within seconds, followed by intracellular edema at 20–30 min. Irreversible injury to myocytes occurs at 30–60 min, and by 60–90 min, vascular endothelial cells sustain damage, leading to necrosis and apoptosis [3]. From a pathophysiological point of view, myocardial viability denotes a state where the myocyte is not irrevocably damaged and retains cellular, metabolic, and contractile function [4,5]. Studies have demonstrated that viable myocardium may be hypocontractile in the setting of chronic myocardial hypoperfusion [3]. In the clinical context, the term refers to dysfunctional myocardium with the potential to regain function if normal blood supply is restored [6].

**Figure 1:**
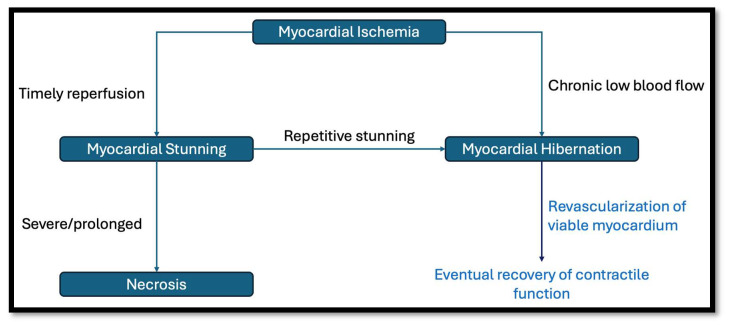
This schematic illustrates the pathophysiological progression of myocardial ischemia and its relation to myocardial viability. Following ischemia, timely reperfusion prevents irreversible damage and leads to myocardial stunning, a reversible condition characterized by prolonged contractile dysfunction despite restored blood flow. Chronic low-flow states can lead to metabolic adaptations where cardiomyocytes retain their metabolic functions but do not have normal contractile functions. Revascularization in such cases can restore adequate blood flow, enabling hibernating myocardium to recover its contractile function over time. In cases of severe or prolonged ischemia without timely reperfusion, necrosis ensues, marking irreversible myocyte damage.

Myocardial viability hinges on the concepts of myocardial stunning and hibernation (Figure 1). Myocardial stunning is defined as a prolonged, post-ischemic ventricular dysfunction that occurs after brief periods of non-lethal ischemia characterized by reversible contractile dysfunction that persists despite the restoration of adequate blood flow [7]. Experimental studies on animals have shown significantly delayed restoration of myocardial function despite only short periods of ischemia [8].

Myocardial hibernation refers to a region supplied by an atherosclerotic coronary artery that receives enough blood flow to maintain cellular metabolic processes but insufficient to sustain normal contractile function [7]. This concept of a hibernating myocardium was popularized in 1989 by Rahimtoola [9] and was differentiated as a distinct entity from myocardial stunning by Conti in 1991 [10]. In their original paper in 1982, Braunwald and Kloner postulated that repetitive episodes of ischemia could result in chronic myocardial stunning [2].

Animal models have demonstrated that myocardial hibernation involves adaptive metabolic changes to mitigate ischemic damage. In a partial stenosis model, myocardial lactate consumption shifted to lactate production in the early minutes of placing a stenosis on a coronary artery but reverted to lactate consumption within a couple of hours [11]. Controversy exists regarding whether hibernation results from chronically reduced resting coronary flow or repetitive episodes of ischemia and stunning [12,13]. Metabolic adaptations such as a shift towards glucose metabolism were postulated to be a transition marker from myocardial stunning to hibernation in an experimental pig model [14]. Furthermore, studies using a chronic ameroid constrictor model in pigs showed that repeated stunning can progressively reduce myocardial blood flow, creating a continuum from normal flow restoration to reduced resting flow seen in hibernation, bridging the gap between the two theories [15,16]. Clinical observations further support a mismatch between flow and function in patients with wall motion abnormalities [17].

The postulated molecular mechanisms responsible for myocardial hibernation are succinctly summarized by Trimarchi et al. and include molecular adaptations such as cellular dedifferentiation, changes in structure and regulation of myofilament proteins, calcium handling abnormalities such as decreased sarcoendoplasmic reticulum calcium ATPase expression, metabolic shifts favoring glucose utilization over fatty acid oxidation, mitochondrial dysfunction affecting efficient ATP production, and oxidative stress. Additionally, downregulation of α- and β-adrenergic receptors, increased apoptotic signaling, and gap junction disruption can further perpetuate dysfunction [18].

### 1.2. Assessing Myocardial Viability

The concept of hibernating myocardium in ischemic heart failure has led to increasing interest in preoperative evaluations for myocardial viability before revascularization. While there is a wealth of observational literature about viability testing in predicting improvement in global LV dysfunction following revascularization, there is no established consensus on the best clinical approach for testing hibernation. As a result, various imaging techniques have been employed, including morphology-based methods such as Cardiac Magnetic Resonance Imaging (CMRI) and echocardiography, perfusion-based approaches using CMRI and Single-Photon Emission Computed Tomography (SPECT), and techniques to evaluate metabolic function such as PET (Positron Emission Tomography) [19]. The literature behind viability testing is heterogenous, with differing specificity and sensitivity in detecting the presence of hibernating myocardium. In a meta-analysis of 158 studies, dobutamine stress echocardiography had the highest specificity and positive predictive values while PET had the highest sensitivity and negative predictive values [20]. We discuss briefly here the different modalities commonly used to assess for myocardial viability. These modalities are briefly summarized in Table 1.

#### 1.2.1. SPECT/PET

SPECT is often combined with PET to assess myocardial viability. SPECT is mainly used to evaluate perfusion, whereas PET is used to evaluate cellular metabolism. 18F-Fluorodeoxyglucose (18F-FDG) is a glucose analog that is used in viability testing as glucose uptake is a good marker for myocardial metabolism [23]. SPECT using 99m-technicium-sestamibi (^99m^Tc-MIBI) as a radiotracer offers better resolution and a lower radiation dose than the previously used thallium-201 (^201^Tl) [19]. Areas of mismatch, where low blood flow is identified using SPECT imaging with ^99m^Tc-labeled tetrofosmin, but high metabolism is observed using 18F-fluorodeoxyglucose PET (FDG-PET), indicate viable but hypoperfused myocardial tissue. In contrast, regions showing both reduced FDG uptake and diminished blood flow (classified as a match) are considered non-viable tissue [24].

#### 1.2.2. Cardiac Magnetic Resonance Imaging

**Figure 2:**
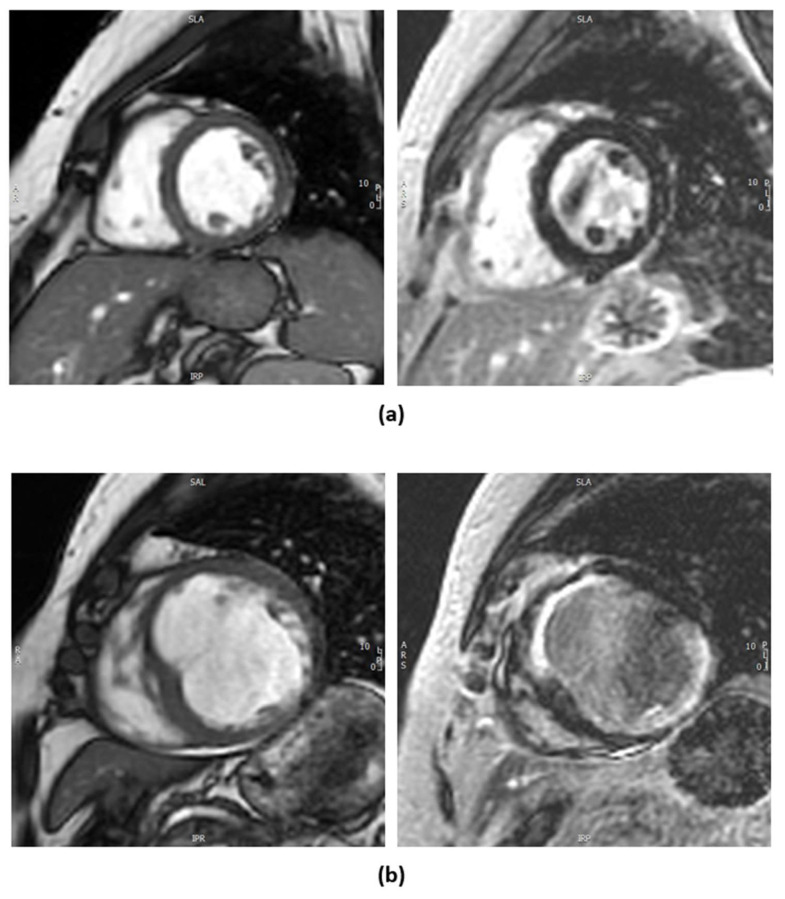
Contrast cardiac MRI with gadolinium contrast. (**a**) Left image shows a 2D cine image clip of mid-ventricle short axis of a patient with ischemic cardiomyopathy. The image to the right corresponds to post-gadolinium contrast imaging showing no late gadolinium enhancement (LGE) indicating viable myocardium. (**b**) Left image shows a 2D cine image clip of mid-ventricle short axis of another patient. There is thinning of the anteroseptal, anterior, and inferolateral segments. The right image corresponds to post-gadolinium imaging with full-thickness LGE in the anterior, anteroseptal, and inferolateral segments indicating non-viable myocardium in the left anterior descending artery (LAD) and left circumflex (LCx) territories.

There are two methods of myocardial viability testing by CMRI. One involves dobutamine to assess for contractile reserve and the other is the evaluation of late gadolinium enhancement (LGE) looking into the volume of distribution of gadolinium-based contrast agents in the extracellular space, which is inversely related to the proportion of viable myocardial cells (Figure 2), with the latter as the more preferred and commonly used technique for assessing viability [21]. In a canine model, LGE-CMRI reliably differentiated between reversible and irreversible myocardial injury, with hyperenhancement corresponding to regions of necrosis or fibrosis and the absence of hyperenhancement indicating viable myocardium regardless of wall motion abnormalities [25]. Scar within the myocardium has significant prognostic implications as it implies irreversible injury and does not lead to reversible LV dysfunction; however, regionally thinned myocardium (defined as an end-diastolic wall thickness of ≤5.5 mm) [6], often misinterpreted as non-viable scar tissue, may recover in contractility and wall thickness after revascularization if the scar burden is limited (≤50% transmural extent of infarction) [26]. However, the availability and high cost of CMRI can be limiting factors, and its use requires significant expertise.

#### 1.2.3. Dobutamine Stress Echocardiography

Echocardiography not only evaluates contractility and wall thickness at rest but also assesses myocardial viability and contractile reserve when combined with low-dose dobutamine. Criteria derived from dobutamine stress echocardiography (DSE) have proven highly effective in predicting regional and global left ventricular functional recovery, both in the acute phase following myocardial infarction and in patients undergoing chronic coronary revascularization [3]. Dobutamine is started at a low dose and gradually uptitrated with the addition of atropine as needed to achieve at least 85% of maximal predicted heart rate (220-age) [27]. A meta-analysis showed low-dose dobutamine stress echocardiography achieved a positive predictive value of 83% and a negative predictive value of 81% in predicting the recovery of contractile function following coronary revascularization [28]. Traditionally, the presence of contractile reserve in a minimum of >5 segments is linked to functional recovery after revascularization [29]. However, with recent clinical trials, this has been called into question [30,31,32]. Some argue that assessing global contractile reserve using an ejection fraction (EF) and wall motion score provides a more comprehensive and accurate evaluation of myocardial viability, as it incorporates the extent and magnitude of left ventricular dysfunction, which are thought to be better predictors of prognosis and outcomes following revascularization [33].

Advances in echocardiographic technology, such as speckle-tracking echocardiography (STE), enable strain measurement, offering a more objective and reproducible assessment of myocardial function compared to traditional wall motion analysis. However, its limitations include clinical factors, such as hemodynamic variability, and technical challenges, including inter-vendor differences in post-processing algorithms, which can affect measurement consistency and interpretation [34]. Small studies have investigated the combination of DSE and speckle-tracking strain analysis in predicting myocardial contractile recovery after revascularization in patients with ischemic cardiomyopathy. A meta-analysis of nine prospective trials including 525 patients demonstrated that low-dose dobutamine (LDD) combined with longitudinal or circumferential strain analysis had high sensitivity (81.5%) and specificity (81.3%) in detecting reversible myocardial dysfunction. However, the findings were limited by small sample sizes, variability in study methodologies, and differing definitions of myocardial viability [35].

### 1.3. Does the Presence of Myocardial Viability Translate to Better Outcomes Following Revascularization?

In 2002, a meta-analysis by Allman KC et al. showed that the presence of myocardial viability significantly predicts benefit following myocardial revascularization vs. optimal medical therapy (16% vs. 3.2%. *p* < 0.0001) [36]. However, the studies included in the meta-analysis were largely retrospective and observational in design with a heterogenous methodology and definition of myocardial viability. Moreover, the studies that were included were before the advent of modern guideline-directed medical therapy (GDMT) with little information in the reports on background medical therapy.

A randomized controlled trial that explored any improvement in outcomes if revascularization was guided by viability using PET reported no difference in the composite primary endpoints of cardiac death, myocardial infarction (MI), or hospitalization for unstable angina or heart failure (HR 0.82, 95% CI 0.62–1.07, *p* = 0.15) and no difference in the secondary hard endpoints of cardiac death (HR 0.80, 95% CI 0.45–1.43, *p* = 0.46) and MI (HR 1.67, 95% CI 0.69–4.01, *p* = 0.25) over a 5-year follow-up among patients with severe LV dysfunction and suspected coronary artery disease (CAD) who were candidates for revascularization with either percutaneous coronary intervention (PCI) or Coronary Artery Bypass Grafting (CABG). However, 25% of patients did not adhere to PET recommendations [32,37]. Although there were marginal statistically significant improved outcomes for those who adhered to PET recommendations, interpretation of this finding is marred by a small study sample size that were adherent to PET recommendations (142 patients vs. 392 patients) and the lack of impact of viability-guided management to major hard endpoints (all-cause death and cardiac death) [32,38].

The STITCH trial published in 2011 randomized 1212 patients with severe LV dysfunction amenable to CABG + optimal medical therapy (OMT) and OMT alone. The long-term follow-up findings of this study demonstrated that CABG was associated with a lower risk of all-cause mortality through a median follow-up of 10.4 years, with an adjusted hazard ratio (HR) of 0.73 (95% CI 0.60–0.90; *p* < 0.01) when compared to medical therapy alone. A prespecified sub-study of the STICH trial, which included 601 patients who underwent SPECT and/or DSE, revealed that this benefit was observed regardless of myocardial viability. Mortality rates were 64% in patients with viable myocardium and 68% in those without viable myocardium (HR 0.81; 95% CI 0.63–1.03; *p* = 0.09). Additionally, the presence of viability did not significantly influence the treatment effect of CABG compared to medical therapy alone (*p* = 0.34 for interaction). The mechanism of benefit is thought to involve the prevention of future fatal myocardial infarctions and the stabilization of arrhythmic substrates [31,39,40]. Another interesting finding of this study was that the presence of viable myocardium was associated with improved ejection fraction regardless of CABG or medical management, highlighting the efficacy of current guideline-based medical therapy.

The recent REVIVED-BCIS2 trial was a randomized clinical trial that investigated the difference in outcomes between revascularization with percutaneous coronary intervention (PCI) or optimal medical therapy (OMT) in patients with severe ischemic cardiomyopathy and extensive coronary artery disease (British Cardiovascular Intervention Society score of 6 or more). In the prespecified secondary analysis of 610 patients with evidence of myocardial viability on dobutamine stress echocardiography or cardiac MRI, the primary outcome (a composite of all-cause mortality or hospitalization for heart failure) occurred in 36.3% of patients in the PCI group and 36.2% in the OMT group (HR 0.99; 95% CI 0.76–1.29; *p* = 0.93), demonstrating no significant difference between the two groups. Similarly, no association was found between the extent of viable myocardium and the primary outcome (HR per 10% increase: 0.98; 95% CI 0.93–1.04; *p* = 0.56). There was improvement in LV function at 6 months regardless of assignment to the PCI or OMT arm of the study, which was associated with improved patient outcomes (HR for every 5% increase in LVEF: 0.87; 95% CI: 0.79–0.95; *p* = 0.003). The presence of extensive scar tissue was associated with a lower likelihood of LV functional improvement (odds ratio per 10% increase in scar: 0.69; 95% CI: 0.56–0.84; *p* < 0.001) [30].

A summary of these recent trials is found in Table 2. Overall, the generalizability of the findings of these trials is limited by variations in modalities and definitions of myocardial viability. Different modalities assess viability differently—SPECT/PET identifies hypoperfused myocardium with intact metabolic function, while dobutamine stress echocardiography evaluates contractile reserve. The optimal way of assessing viability that is meaningful in terms of patient long-term outcomes seems to be a dynamic field of study with some experts proposing combining PET and CMR to assess for myocardial viability [41,42].

Major international guidelines have started to incorporate the findings of these trials into their position statements. For example, the latest joint European Society of Cardiology and European Association for Cardiothoracic Surgery (ESC/EACTS), which was published in 2018, emphasized using viability testing only in conjunction with other clinical variables [43]. The more recent American College of Cardiology/American Heart Association (ACC/AHA) position statement, published in 2021, discouraged routine viability testing, highlighting the uncertainty in its impact on guiding treatment decisions and the assessment method that would provide the most useful information [44].

## 2. Limitations

While this paper provides a comprehensive review of myocardial viability assessment, several limitations must be acknowledged. This is a narrative review and does not follow a structured methodology for the literature selection. Therefore, although the literature search has been vetted by the three authors, including two senior interventional cardiologists and a cardiac imaging specialist, there might be potential biases in the literature selection. Additionally, the literature is inherently heterogeneous with studies utilizing different techniques and definitions for myocardial viability. This lack of uniformity limits the direct comparability of the trial results.

### Future Directions

The integration of complementary imaging modalities is a promising direction for myocardial viability assessment. The limitations of individual techniques have driven interest in hybrid approaches that combine the strengths of different modalities, such as PET-MRI [42]. Other novel approaches, such as myocardial work assessment—which integrates left ventricular pressure measurements into strain calculations to overcome the load dependency limitations of traditional parameters like LVEF and global longitudinal strain—are also an evolving area of investigation [45].

Future research should prioritize large, prospective randomized controlled trials to clarify the role of viability testing in guiding revascularization decisions in the era of modern guideline-directed medical therapy. Standardizing consensus criteria for what constitutes viable myocardium would enable more meaningful comparisons across studies. Additionally, the advent of artificial intelligence and modern machine learning algorithms could enhance the interpretation of complex multimodal imaging data, reduce inter-observer variability, and represent a new frontier in the application of these technologies.

## 3. Conclusions

Advances in imaging modalities, including PET, SPECT, cardiac MRI, and dobutamine stress echocardiography, have enhanced our ability to identify viable myocardium, predict functional recovery, and guide revascularization strategies. However, recent evidence from large clinical trials such as STICH and REVIVED-BCIS2 challenges the traditional reliance on viability testing for guiding revascularization, showing no consistent association between viability and improved outcomes in terms of mortality or heart failure hospitalizations. Interestingly, OMT alone was shown to improve left ventricular function, and this improvement was associated with better patient outcomes. These findings underscore the increasing importance of guideline-directed medical therapy and suggest that viability assessment should be individualized, particularly for patients in whom the benefit of revascularization remains uncertain, as part of a broader, multifaceted treatment approach.

## Figures and Tables

**Table 1:** Summary of common modalities to assess myocardial viability [6,19,21,22].

Modality	Key Features	Advantages	Limitations
SPECT/PET	SPECT evaluates perfusion; PET measures glucose metabolism. Mismatch between perfusion and metabolism suggests viability.	High sensitivity for viability. Assesses for presence of intact cellular metabolism.	Ionizing radiation. PET less available due to infrastructure needs (e.g., cyclotron for some tracers). SPECT has lower spatial resolution.
Cardiac MRI (CMRI)	Identifies scar burden by measuring contrast uptake in extracellular space.	High spatial resolution. No ionizing radiation.	Expensive. Requires expertise. Not suitable for all patients (e.g., claustrophobia, metal implants).
Dobutamine Stress Echo	Evaluates contractile reserve by assessing wall motion response to stress.	Widely available. No radiation. Lower cost.	Operator-dependent. Limited spatial resolution. Challenging in poor acoustic windows.

**Table 2:** Summary of key findings from three major trials evaluating myocardial viability and its impact on outcomes in patients undergoing revascularization.

Trial	PARR-2 (Long-Term Follow-Up)	STITCH (Long-Term Follow-Up)	REVIVED-BCIS2
Patient numbers	392 patients for long term follow-up (197 PET-assisted vs. 195 standard care)	1212 with 601 undergoing viability testing	700 patients. 610 patients with LVEF ≤ 35%, extensive coronary artery disease and evidence of viability in at least 4 myocardial segments
Viability testing modality	FDG-PET	SPECT or DSE	CMRI or DSE
Mean LVEF at enrollment	27%	28%	27%
Primary outcome	Cardiovascular death, MI, hospital admission due to cardiac cause	All-cause mortality	All-cause mortality, heart failure hospitalization
Secondary outcomes	Time to primary outcome, time to cardiovascular death	Cardiovascular death, death by any cause, hospitalization for cardiac cause	All-cause death, cardiovascular death, heart failure hospitalization, and improved LV function at 6 months
Revascularization technique	CABG and PCI	CABG	PCI
Median follow-up	5 years	10.4 years	3.4 years

## Data Availability

Not applicable.

## Abbreviations

LV	Left ventricle
CMRI	Cardiac Magnetic Resonance Imaging
SPECT	Single-Photon Emission Computed Tomography
PET	Positron Emission Tomography
18F-FDG	18F-Fluorodeoxyglucose
LAD	Left anterior descending artery
LCx	Left circumflex artery
^99m^Tc-MIBI	99m-technicium-sestamibi
LGE	Late gadolinium enhancement
DSE	Dobutamine stress echocardiography
STE	Speckle-tracking echocardiography
LDD	Low-dose dobutamine
EF	Ejection fraction
MI	Myocardial infarction
GDMT	Guideline-directed medical therapy
CAD	Coronary artery disease
PCI	Percutaneous coronary intervention
CABG	Coronary Artery Bypass Grafting
OMT	Optimal medical therapy

## References

[1] Savarese G., Becher P.M., Lund L.H., Seferovic P., Rosano G.M.C., Coats A.J.S. (2023). Global burden of heart failure: A comprehensive and updated review of epidemiology. Cardiovasc. Res..

[2] Braunwald E., Kloner R.A. (1982). The stunned myocardium: Prolonged, postischemic ventricular dysfunction. Circulation.

[3] Jamiel A., Ebid M., Ahmed A.M., Ahmed D., Al-Mallah M.H. (2017). The Role of Myocardial Viability in Contemporary Cardiac Practice.

[4] Gunning M.G., Kaprielian R.R., Pepper J., Pennell D.J., Sheppard M.N., Severs N.J., Underwood S.R. (2002). The histology of viable and hibernating myocardium in relation to imaging characteristics. J. Am. Coll. Cardiol..

[5] Ryan M., Morgan H., Chiribiri A., Nagel E., Cleland J., Perera D. (2022). Myocardial Viability Testing: All Stiched Up, or About to Be Revived?.

[6] Garcia M.J., Kwong R.Y., Scherrer-Crosbie M., Taub C.C., Blankstein R., Lima J., Bonow R.O., Eshtehardi P., Bois J.P. (2020). State of the Art: Imaging for Myocardial Viability: A Scientific Statement from the American Heart Association. Circ. Cardiovasc. Imaging.

[7] Kloner R.A. (2020). Stunned and Hibernating Myocardium: Where Are We Nearly 4 Decades Later?. J. Am. Heart Assoc..

[8] Weil B.R., Young R.F., Shen X., Suzuki G., Qu J., Malhotra S., Canty J.M. (2017). Brief Myocardial Ischemia Produces Cardiac Troponin I Release and Focal Myocyte Apoptosis in the Absence of Pathological Infarction in Swine. JACC. Basic Transl. Sci..

[9] Rahimtoola S.H. (1989). The hibernating myocardium. Am. Heart J..

[10] Conti C.R. (1991). The stunned and hibernating myocardium: A brief review. Clin. Cardiol.

[11] Fedele F.A., Gewirtz H., Capone R.J., Sharaf B., Most A.S. (1988). Metabolic response to prolonged reduction of myocardial blood flow distal to a severe coronary artery stenosis. Circulation.

[12] Ryan M.J., Perera D. (2018). Identifying and Managing Hibernating Myocardium: What’s New and What Remains Unknown?. Curr. Heart Fail. Rep..

[13] Kim S.J., Peppas A., Hong S.K., Yang G., Huang Y., Diaz G., Vatner S. (2003). Persistent stunning induces myocardial hibernation and protection: Flow/function and metabolic mechanisms. Circ. Res..

[14] Fallavollita J.A., Canty J.M. (1999). Differential 18 F-2-Deoxyglucose Uptake in Viable Dysfunctional Myocardium with Normal Resting Perfusion Evidence for Chronic Stunning in Pigs. Circulation.

[15] Canty J.M., Fallavollita J.A. (2000). Chronic hibernation and chronic stunning: A continuum. J. Nucl. Cardiol..

[16] Canty J.M., Fallavollita J.A. (2001). Lessons from experimental models of hibernating myocardium. Coron. Artery Dis..

[17] Nihoyannopoulos P., Vanoverschelde J.L. (2011). Myocardial ischaemia and viability: The pivotal role of echocardiography. Eur. Heart J..

[18] Trimarchi G., Trimarchi G., Teresi L., Licordari R., Pingitore A., Pizzino F., Grimaldi P., Di Bella G. (2024). Transient Left Ventricular Dysfunction from Cardiomyopathies to Myocardial Viability: When and Why Cardiac Function Recovers. Biomedicines.

[19] Madsen S., Dias A.H., Lauritsen K.M., Bouchelouche K., Tolbod L.P., Gormsen L.C. (2020). Myocardial Viability Testing by Positron Emission Tomography: Basic Concepts, Mini-Review of the Literature and Experience From a Tertiary PET Center. Semin. Nucl. Med..

[20] Schinkel A.F.L., Bax J.J., Poldermans D., Elhendy A., Ferrari R., Rahimtoola S.H. (2007). Hibernating Myocardium: Diagnosis and Patient Outcomes. Curr. Probl. Cardiol..

[21] Al-Sabeq B., Nabi F., Shah D.J. (2019). Assessment of myocardial viability by cardiac MRI. Curr. Opin. Cardiol..

[22] Ker W.D.S., Nunes T.H.P., Nacif M.S., Mesquita C.T. (2018). Practical implications of myocardial viability studies. Arq. Bras. Cardiol..

[23] Wende A.R., Brahma M.K., McGinnis G.R., Young M.E. (2017). Metabolic Origins of Heart Failure. JACC Basic Transl. Sci..

[24] Lehtinen M., Schildt J., Ahonen A., Nikkinen P., Lauerma K., Sinisalo J., Pöyhiä R. (2015). Combining FDG-PET and 99mTc-SPECT topredict functional outcome after coronary artery bypass surgery. Eur. Heart J. Cardiovasc. Imaging.

[25] Kim R.J., Fieno D.S., Parrish T.B., Harris K., Chen E.L., Simonetti O., Bundy J., Finn J.P., Klocke F.J., Judd R.M. (1999). Relationship of MRI Delayed Contrast Enhancement to Irreversible Injury, Infarct Age, and Contractile Function. Circulation.

[26] Shah D.J., Kim H.W., James O., Parker M., Wu E., Bonow R.O., Kim R.J. (2013). Prevalence of regional myocardial thinning and relationship with myocardial scarring in patients with coronary artery disease. JAMA.

[27] Pellikka P.A., Arruda-Olson A., Chaudhry F.A., Chen M.H., Marshall J.E., Porter T.R., Sawada S.G. (2020). Guidelines for Performance, Interpretation, and Application of Stress Echocardiography in Ischemic Heart Disease: From the American Society of Echocardiography. J. Am. Soc. Echocardiogr..

[28] Bonow R.O. (1996). Identification of viable myocardium. Circulation.

[29] La Canna G., Alfieri O., Giubbini R., Gargano M., Ferrari R., Visioli O. (1994). Echocardiography during infusion of dobutamine for identification of reversible dyfunction in patients with chronic coronary artery disease. J. Am. Coll. Cardiol..

[30] Perera D., Ryan M., Morgan H.P., Greenwood J.P., Petrie M.C., Dodd M., Weerackody R., O’Kane P.D., Masci P.G., Nazir M.S. (2023). Viability and Outcomes with Revascularization or Medical Therapy in Ischemic Ventricular Dysfunction: A Prespecified Secondary Analysis of the REVIVED-BCIS2 Trial. JAMA Cardiol..

[31] Panza J.A., Ellis A.M., Al-Khalidi H.R., Holly T.A., Berman D.S., Oh J.K., Pohost G.M., Sopko G., Chrzanowski L., Mark D.B. (2019). Myocardial Viability and Long-Term Outcomes in Ischemic Cardiomyopathy. N. Engl. J. Med..

[32] Ardle B.M., Shukla T., Nichol G., deKemp R.A., Bernick J., Guo A., Beanlands R.S. (2016). Long-Term Follow-Up of Outcomes with F-18-Fluorodeoxyglucose Positron Emission Tomography Imaging-Assisted Management of Patients with Severe Left Ventricular Dysfunction Secondary to Coronary Disease. Circ. Cardiovasc. Imaging.

[33] Khemka A., Sawada S.G. (2019). Dobutamine echocardiography for assessment of viability in the current era. Curr. Opin. Cardiol..

[34] Collier P., Phelan D., Klein A. (2017). The Present and Future Review Topic of The Week a Test in Context: Myocardial Strain Measured by Speckle-Tracking Echocardiography. J. Am. Coll. Cardiol.

[35] Ballo H., Doghman F., Hartikainen J., Saraste A., Knuuti J. (2023). Speckle-tracking echocardiography for predicting improvement of myocardial contractile function after revascularization: A meta-analysis of prospective trials. Int. J. Cardiovasc. Imaging.

[36] Allman K.C., Shaw L.J., Hachamovitch R., Udelson J.E. (2002). Coronary Revascularization Myocardial Viability Testing and Impact of Revascularization on Prognosis in Patients with Coronary Artery Disease and Left Ventricular Dysfunction: A Meta-Analysis. J. Am. Coll. Cardiol..

[37] Beanlands R.S., Nichol G., Huszti E., Humen D., Racine N., Freeman M., PARR-2 Investigators (2007). F-18-Fluorodeoxyglucose Positron Emission Tomography Imaging-Assisted Management of Patients with Severe Left Ventricular Dysfunction and Suspected Coronary Disease: A Randomized, Controlled Trial (PARR-2). J. Am. Coll. Cardiol..

[38] Liga R., Colli A., Taggart D.P., Boden W.E., De Caterina R. (2023). Myocardial Revascularization in Patients with Ischemic Cardiomyopathy: For Whom and How. J. Am. Heart Assoc..

[39] Velazquez E.J., Lee K.L., Deja M.A., Jain A., Sopko G., Marchenko A., Rouleau J.L. (2011). Coronary-Artery Bypass Surgery in Patients with Left Ventricular Dysfunction. N. Engl. J. Med..

[40] STICH Myocardial Viability at 10 Years: Still No Link with CABG Effects|tctmd.com. https://www.tctmd.com/news/stich-myocardial-viability-10-years-still-no-link-cabg-effects.

[41] Allman K.C. (2013). Noninvasive assessment myocardial viability: Current status and future directions. J. Nucl. Cardiol..

[42] Whittington B., Dweck M.R., van Beek E.J.R., Newby D., Williams M.C. (2023). PET-MRI of Coronary Artery Disease. J. Am. Coll. Cardiol.

[43] Neumann F.J., Sousa-Uva M., Ahlsson A., Alfonso F., Banning A.P., Benedetto U., Zembala M.O. (2019). 2018 ESC/EACTS Guidelines on myocardial revascularization. Eur. Heart J..

[44] Lawton J.S., Tamis-Holland J.E., Bangalore S., Bates E.R., Beckie T.M., Zwischenberger B.A. (2022). 2021 ACC/AHA/SCAI Guideline for Coronary Artery Revascularization: A Report of the American College of Cardiology/American Heart Association Joint Committee on Clinical Practice Guidelines. J. Am. Coll. Cardiolog..

[45] Trimarchi G., Carerj S., Di Bella G., Manganaro R., Pizzino F., Restelli D., Zito C. (2024). Clinical Applications of Myocardial Work in Echocardiography: A Comprehensive Review. J. Cardiovasc. Echogr..

